# *g*-C_3_N_4_–Co_3_O_4_ Z-Scheme Junction with Green-Synthesized ZnO Photocatalyst for Efficient Degradation of Methylene Blue in Aqueous Solution

**DOI:** 10.1155/2023/2948342

**Published:** 2023-06-05

**Authors:** Mintesinot Tamiru Mengistu, Tadele Hunde Wondimu, Dinsefa Mensur Andoshe, Jung Yong Kim, Osman Ahmed Zelekew, Fekadu Gashaw Hone, Newaymedhin Aberra Tegene, Noto Susanto Gultom, Ho Won Jang

**Affiliations:** ^1^Department of Materials Science and Engineering, Adama Science and Technology University, P.O. Box 1888, Adama, Ethiopia; ^2^Center of Advanced Materials Science and Engineering, Adama Science and Technology University, P.O. Box 1888, Adama, Ethiopia; ^3^Physics Department, Addis Ababa University, Addis Ababass 1176, Ethiopia; ^4^Department of Materials Science and Engineering, National Taiwan University of Science and Technology, Taipei 10607, Taiwan; ^5^Department of Materials Science and Engineering Research Institute of Advanced Materials Seoul National University, Seoul 08826, Republic of Korea

## Abstract

A simple wet chemical ultrasonic-assisted synthesis method was employed to prepare visible light-driven g-C_3_N_4_-ZnO-Co_3_O_4_ (GZC) heterojunction photocatalysts. X-ray diffraction (XRD), scanning electromicroscopy (SEM), Fourier-transform infrared spectroscopy (FTIR), Brunauer–Emmett–Teller (BET), ultraviolet (UV), and electrochemical impedance spectroscopy (EIS) are used to characterize the prepared catalysts. XRD confirms the homogenous phase formation of g-C_3_N_4_, ZnO, and Co_3_O_4_, and the heterogeneous phase for the composites. The synthesized ZnO and Co_3_O_4_ by using cellulose as a template show a rod-like morphology. The specific surface area of the catalytic samples increases due to the cellulose template. The measurements of the energy band gap of a g-C_3_N_4_-ZnO-Co_3_O_4_ composite showed red-shifted optical absorption to the visible range. The photoluminescence (PL) intensity decreases due to the formation of heterojunction. The PL quenching and EIS result shows that the reduction of the recombination rate and interfacial resistance result in charge carrier kinetic improvement in the catalyst. The photocatalytic performance in the degradation of MB dye of the GZC-3 composite was about 8.2-, 3.3-, and 2.5-fold more than that of the g-C_3_N_4_, g-C_3_N_4_-ZnO, and g-C_3_N_4_-Co_3_O_4_ samples. The Mott–Schottky plots of the flat band edge position of g-C_3_N_4_, ZnO, Co_3_O_4_, and Z-scheme g-C_3_N_4_-ZnO-Co_3_O_4_ photocatalysts may be created. Based on the stability experiment, GZC-3 shows greater photocatalytic activity after four recycling cycles. As a result, the GZC composite is environmentally friendly and efficient photocatalyst and has the potential to consider in the treatment of dye-contaminated wastewater.

## 1. Introduction

Today's society faces environmental challenges due to the ever-increasing demand for energy, the excessive use of fossil fuels, and related complications such as greenhouse gas emissions. Organic dyes are chemical pollutants that contaminate water. These contaminants in wastewater are very hazardous, can cause carcinogens, and are dangerous to humans, animals, and entire ecosystems [1]. There are approximately 10,000 varieties of commercially used dyes, with an annual production of around 0.7 billion tons [2]. About 20% of these colors are lost during dyeing processes and discharged as textile effluents [3]. Various methods, in particular, ion exchange [4], coagulation-flocculation [5], oxidation [6], electrochemical treatment [7], and membrane-based filters have been employed in the removal of organic contaminants from polluted solution [8].

Photocatalysis has emerged as an intriguing degradation mechanism among the existing potential methods of removing organic effluents from the solution due to its low cost, nontoxicity, safety, and renewable nature. TiO_2_, which was used in 1972 by Fujishima for photoelectrochemical water splitting, is the most extensively used photocatalyst today [9]. The typical TiO_2_ catalyst, on the other hand, is stimulated by UV light that takes up less than 5% of the whole solar spectrum. This has prompted researchers to create innovative materials with lower bandgap energy (*E*_g_) to improve sensitivity to an increasingly plentiful visible light photon [10–15]. Therefore, the heterojunction of cobalt(II, III) oxide and bismuth oxyiodides efficiently remove nitrophenol from solutions due to the high specific surface area and density of the photogenerated charge carrier resulting in its visible light-sensitivity of the catalyst [16, 17]. Acid Blue 25 dyes were removed effectively by tetraphenylporphyrin/tungsten (VI) oxide/reduced graphene oxide photocatalyst [18]. Moreover, mesoporous dendritic silica supports TiO_2_ to improve the catalytic performance for the degradation of carbamazepine due to the reduction of the charge recombination rate, charge kinetics improvement, and increased density of the active site for the analyte adsorption [19].

Graphitic carbon nitride (g-C_3_N_4_) has recently gained a lot of interest because it possesses ∏-conjugated planar layers akin to graphite, giving it great thermal and chemical stability as well as an attractive electrical structure [20]. As a result, it could be used as a direct semiconductor catalyst in sustainable chemistry. The g-C_3_N_4_ has distinct properties such as a good electrical and optical structure, as well as strong photochemical stability, and is considered a potential photocatalyst [21–23]. g-C_3_N_4_ has been shown to function well in photo-degrading various organic dyes when exposed to visible light [24, 25]. Nevertheless, due to its small surface area, limited visible light absorption, and the electron-hole recombination rate, the photocatalytic efficiency of g-C_3_N_4_ is far from optimal. Several ways have been used to overcome these limitations. Many methods, including chemical and physical exfoliation methods, have been used to enhance the surface area of g-C_3_N_4_. Exfoliation of g-C_3_N_4_ provides not only a high surface area but also a shorter diffusion length which might result in a low recombination rate [26–29]. Doping with metals and nonmetals has also been found to be an effective method for increasing visible light absorption using bandgap engineering [30]. Qiao's group demonstrated that phosphorus doping porous g-C_3_N_4_ nanosheets can significantly lower the bandgap from 2.98 to 2.66 eV which creates more photo-excitation of electrons and holes [31–34]. Concerning the high electron-hole recombination rate of g-C_3_N_4_, it can be reduced by loading noble metals on its surface or creating junction with different other semiconductors such as TiO_2_/g-C_3_N_4_ [35], graphene oxide/g-C_3_N_4_ [36], Au/g-C_3_N_4_ [37]/g-C_3_N_4_/*α*-Fe_2_O_3_ [38], g-C_3_N_4_/Ag_2_O/TiO_2_ [39], g-C_3_N_4_/Bi_2_WO_6_ [40], Ag/g-C_3_N_4_ [41], g-C_3_N_4_/Co_3_O_4_/V_2_O_5_ [42], Cu doped ZnO/g-C_3_N_4_ [43], g-C_3_N_4_/Co_3_O_4_ [44], BiOBr-NFs/g-C_3_N_4_-SAF [45], and Co_3_O_4_@CeO_2_ [46], and ZnO is an inexpensive and nontoxic semiconductor material that has been utilized as a photocatalyst; however, due to its high band gap (3.2-3.3 eV), it is only active in the UV region of the solar spectrum [47–49]. Several literatures demonstrate that the ZnO-coupled g-C_3_N_4_ composite has shown to increase the oxidation potential and removal efficiency of inorganic and organic contaminants. However, due to reduced visible light absorption in the solar spectrum, the application of the g-C_3_N_4_-ZnO composite has restricted activity in the visible light area. To solve this issue, a p-type inorganic spinal cobalt oxide (Co_3_O_4_) semiconductor material with great thermal durability, nontoxicity, and excellent optical property energy bandgap (2.1 eV) can be used [50]. Few studies have combined all of the above-mentioned approaches for increasing the photocatalytic properties of g-C_3_N_4_. Herein, the incorporation of Co_3_O_4_ into g-C_3_N_4_ was used to increase absorbance in the visible portion of the solar spectrum. Chemical exfoliation of bulk g-C_3_N_4_ was used to reduce the number of stacked layers while increasing the active surface area. We used cellulose extracted from the local plants as a template for metal oxide synthesis because the hydroxyl groups on cellulose act as efficient hydrophilic substrates for metal oxide nucleation and growth, resulting in rod-like morphology which results in a high surface area. The Z-scheme system is designed to enhance the charge carrier density and collection efficiency of the photocatalyst [51]. As a result, the current article presents a thorough examination of the effects of combining all of the approaches on enhancing the performance of g-C_3_N_4_ regarding methylene blue degradation. Under visible light illumination, the composite outperformed pure g-C_3_N_4_ in photodegradation of methylene blue by over 8.2 times. Using various characterization techniques, the role of each approach in enhancing photocatalytic activity of g-C_3_N_4_-ZnO-Co_3_O_4_ compared to g-C_3_N_4_ is discussed.

## 2. Materials and Methods

### 2.1. Material Characterization

The phase composition and crystallinity of the prepared photocatalyst were measured by using XRD, ShimadzuXRD-7000, with CuK *α* radiation. Fourier-transform infrared (FTIR) analysis was performed using Spectrum 65 FTIR (Perkin Elmer) in the range of 4000−400 cm^−1^ using KBrpellets. The morphologies of the samples were examined by field-emission scanning electron microscopy (FESEM, JSM 6500F, and JEOL). The optical absorption spectra were measured using a Shimadzu 3600 UV–Vis-NIR spectrophotometer in the wavelength range of 200–800 nm using BaSO4 as a reference. Brunauer, Emmett, and Teller (BET), ASAP 2020 HD88 surface area analyzer, was used to measure the specific surface area of the samples by N_2_ adsorption. A spectrophotometer, PE-LS55, USA, a xenon lamp light source, and an exciton wavelength of 326 nm were used to measure the photoluminescence spectra of the sample.

EIS and Mott–Schottky were measured using a three-electrode system with an Autolab PGSTAT 302 N electrochemical test system (Metrohm Autolab B.V.), with Pt wire and Ag/AgCl electrode as the counter and reference electrodes, respectively.

### 2.2. Materials

Enset (*Ensete ventricosum*) fibre was collected from Hawssa, South Ethiopia. Glacial acetic acid (CH₃COOH), sodium hydroxide (NaOH), formic acid (CH₂O₂), 30% hydrogen peroxide (H_2_O_2_), urea (CH₄N₂O), sulfuric acid (H_2_SO_4_), cobalt nitrate hexahydrate (Co (NO_3_)_2_•6H_2_O), zinc nitrate hexahydrate (Zn (NO_3_)_2_•6H_2_O), and methylene blue (C_16_H_18_N_3_SCl) were purchased from Sigma Aldrich. All of the chemicals were utilized as purchased, with no further refining.

### 2.3. Synthesis of Cellulose

Cellulose from enset was synthesized, as shown in Figure S1 [40]. The plant fibre was washed and dried in an electric oven at 80°C to eliminate reaming dust. The raw material was boiled with a 2% NaOH (1 : 40) W/V ratio for 2 hr, then dried in an electric oven at 70°C, and chopped. After the first alkaline treatment, the second alkaline pretreatment was carried out on a hot plate at 90°C for 1.5 hr in 10% NaOH solutions with a 1 : 10 (W/V) solid-to-liquid ratio of the dry material. The resulting pulps were centrifuged, dried, and subsequently treated with 20% of CH_2_O_2_/20% of CH₃COOH/7.5% of H_2_O_2_ (2 : 1 : 2) solution at 90°C temperature of hotplate for 1.5 hr. The dignified pulps were filtered and washed with hot water to separate the cooking liquid (which contains lignin and hemicellulose) from the cellulose. Before the bleaching procedure, the pulps were centrifuged and dried. The pulps were bleached for 30 min at 70°C with 7.5% H_2_O_2_ and 10% NaOH solutions. The pulps were washed several times with distilled water to eliminate residuals before drying at 60°C in an oven. Finally, 5 g cellulose was added to a 250 ml 50% H_2_SO_4_ aqueous solution for 30 min at 60°C before being diluted with distilled water. After centrifuging and washing with distilled water until the pH was neutral, the cellulose gel was ultrasonically dispersed in 1 L of distilled water and stored for later use.

### 2.4. Synthesis of ZnO

A simple chemical precipitation synthesis method was used to prepare ZnO, as shown in Figure S2 [41]. In a nutshell, 0.1 M of zinc nitrate hexahydrate was dissolved in 200 ml of dispersed cellulose solution under vigorous stirring for 0.5 hrs, and then the pH of the solution was adjusted to 10 by adding 1 M NaOH drop wise with continuous stirring for precipitation. Then, the solution was stirred for 2 hr at 60°C and later aged for 24 hr. The precipitate was centrifuged and rinsed with distilled water and ethanol. After washing, the precipitate sample was recovered and dried in an electric oven at 100°C for 2 hrs. The dried sample was then calcined in a muffle furnace at 500°C for 2 hrs. After being ground to a powder, the calcined ZnO was recovered and used in future studies.

### 2.5. Synthesis of Co_3_O_4_

Co_3_O_4_ was prepared by a simple chemical precipitation method, as shown in Figure S3 [42]. In a nutshell, 0.1 M of cobalt nitrate hexahydrate was dissolved in 200 ml of dispersed cellulose solution under vigorous stirring for 0.5 hrs, and then the pH of the prepared solution was raised to 10 by adding 1 M NaOH drop wise with continuous stirring for precipitation. Then, the solution was stirred for 2 hrs at 60°C and then aged for 24 hrs. The precipitate was centrifuged and washed with distilled water and ethanol. After washing, the precipitate sample was recovered and dried in an electric oven at 100°C for 2 hrs. The dried sample was then calcined in a muffle furnace at 400°C for 2 hrs. The calcined Co_3_O_4_ was collected and ground to powder and used in future studies.

### 2.6. Synthesis of g-C_3_N_4_

To synthesize g-C_3_N_4_, a thermal pyrolysis method was used, as shown in Figure S4 [31, 32]. 10 g of urea flake was placed in a crucible tightly wrapped in aluminum foil and heated in a muffle furnace at the rate of 2°C min^−1^ up to 550°C and held for 4 hrs. After naturally cooling to room temperature, a pale-yellow sponge-like powder product was collected. 300 mg of as-prepared powder was mixed in 15 ml of H_2_SO_4_ and vigorously stirred at room temperature for 24 hrs to break the bonds between layered stacked structures of g-C_3_N_4_. This solution was diluted with 400 ml of distilled water and ultrasonicated for 6 hrs to exfoliate the g-C_3_N_4_ layers. The suspension was filtered from the residual solution and washed multiple times with distilled water and ethanol to clean any residual acid from the sample. Finally, at 80°C, the mixture was dried to obtain a g-C_3_N_4_ powder.

### 2.7. Synthesis of the g-C_3_N_4_-ZnO-Co_3_O_4_ Composite

A simple wet chemical ultrasonic-assisted synthesis method was employed to prepare the g-C_3_N_4_-ZnO-Co_3_O_4_ composite, as shown in Figure S5. Typically, different weight ratios of Co_3_O_4_ and ZnO catalyst samples were added to g-C_3_N_4_ in 30 ml of ethanol solution, 5% ZnO and 5% Co_3_O_4_ (GZC-1), 10% ZnO and 10% Co_3_O_4_ (GZC-2), 15% ZnO and 15% Co_3_O_4_ (GZC-3), 20% ZnO and 20% Co_3_O_4_ (GZC-4), and 25% ZnO and 25% Co_3_O_4_ (GZC-5). The mixture was sonicated for 30 min and vigorously stirred for 2 hrs, and the resulting solid was dried for 6 hrs at 70°C. The binary composites, g-C_3_N_4_-ZnO (GZ) and g-C_3_N_4_-Co_3_O_4_ (GC), were prepared following a similar process.

## 3. Results and Discussion

### 3.1. X-Ray Diffraction Analysis

**Figure 1:**
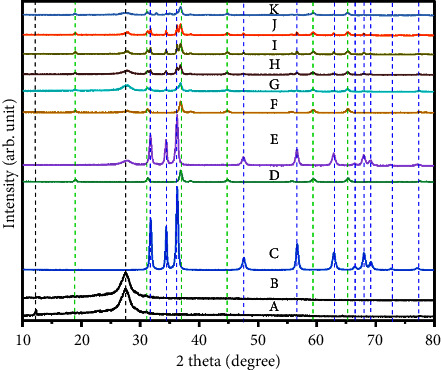
XRD patterns of (A) bulk g-C_3_N_4_, (B) exfoliated g-C_3_N_4_, (C) ZnO, (D) Co_3_O_4_, (E) GZ, (F) GC, (G) GZC-1, (H) GZC-2, (I) GZC-3, (J) GZC-4, and (K) GZC-5.

A simple wet chemical ultrasonic-assisted synthesis method was employed to prepare the g-C_3_N_4_-ZnO-Co_3_O_4_ composite, as shown in Figure S5. XRD was used to examine the phase purity and the crystalline structure of samples.  Figure 1 (A-K) depicts XRD results of bulk g-C_3_N_4_, exfoliated g-C_3_N_4_, Co_3_O_4_, ZnO, g-C_3_N_4_-Co_3_O_4_, g-C_3_N_4_-ZnO, and g-C_3_N_4_-ZnO-Co_3_O_4_.  Figure S6 shows that the XRD result shows peaks at 16°, 22°, and 34° at (2*θ*), respectively, with the assigned crystallographic planes of (110), (200), and (400), indicating the formation of cellulose I, as well as no doublet in the intensity of the peaks, indicating the absence of cellulose II [52]. There are two distinct peaks for bulk g-C_3_N_4_ with a strong signal at 27.5° and 13.2°, which correspond to the (002) and (100) planes of g-C_3_N_4_ (#JCPDS-87-1526) [53] (see Figure 1 A). Tri-s-triazine units and aromatic interlayer stacking are the indication of (002) and (100) crystal planes, respectively [52]. The peak intensity of plane (002) is weak for exfoliated g-C_3_N_4_, as shown in Figure 1 (B), which implies a reduction in the number of stacked layers in g-C_3_N_4_ due to exfoliation using sulfuric acid [41]. The metal oxides XRD result is also depicted in Figure 1 (C and D) (#JCPDS-36-1451) [54]. The diffraction peaks at 31.68°, 34.4°, 36.17°, 47.53°, 56.6°, 62.88°, 64.39°, 68.03°, 69.14°, and 77.03° of 2*θ* belong to (100), (002), (101), (102), (110), (103), (200), (112), (201), (004), and (202) diffraction planes of the wurtzite hexagonal phase of ZnO as shown in Figure 1 (C). The diffraction peaks at 18.9°, 31.6°, 36.8°, 44.8°, 59.3°, and 65.2° of 2*θ* match with the cubic face-centered Co_3_O_4_ crystal planes of (111), (220) (211) (222) (400), (422), (511), (440), and (533), respectively (Figure 1 (D) (#JCPDS-43-1003) [55]. The XRD patterns of g-C_3_N_4_-ZnO, g-C_3_N_4_-Co_3_O_4_, and prepared g-C_3_N_4_-ZnO-Co_3_O_4_ composite catalysts with different weight percent contents of zinc oxide and cobalt oxide to graphitic carbon nitride were illustrated in Figure 1(E-K). The g-C_3_N_4_-ZnO-Co_3_O_4_ composites have matched with original diffraction peaks of g-C_3_N_4_, the wurtzite hexagonal ZnO phase, and the cubic Co_3_O_4_ phase, with no additional peaks present, indicating that the composite has successfully formed. In the composites, a similar peak shape and position as seen in g-C_3_N_4_, ZnO, and Co_3_O_4_ were observed that indicates that the interaction of ZnO and Co_3_O_4_ with g-C_3_N_4_ does not affect the original lattice structure of g-C_3_N_4_.

The diffraction peaks of ZnO and Co_3_O_4_ were steadily strengthened at the cost of the g-C_3_N_4_ peak intensity while the ZnO and Co_3_O_4_ concentrations increased. Furthermore, no additional peaks appeared in the samples for any possible impurities or phases.

### 3.2. FTIR Analysis

**Figure 2:**
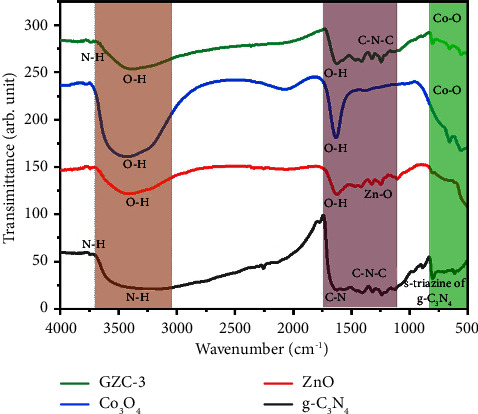
FTIR spectra of g-C_3_N_4_, ZnO, Co_3_O_4_, and GZC-3 composites.

In Figure 2, three separate bands in the FTIR spectra of the g-C_3_N_4_, ZnO, Co_3_O_4_, and GZC-3 samples were observed. The heterocyclic aromatic bonds, C-N, are associated with the stretching vibration of C=N, C-N, and C-N-C. The stretching vibration peak is predicted at the wavenumber range of 1200–1650 cm^−1^, along with 1238, 1318, 1410, 1574, and 1640 cm^−1^ [56–59]. The broad peak in the vicinity of 3000–3500 cm^−1^ is attributed to the N-H stretching of residual amine groups (-NH_2_ and -NH), which might be a residue of the precursor urea. This broad peak might also attribute to the O-H stretching band due to moisture absorption of the sample from the environment [60]. The typical characteristic peaks at around 808 cm^−1^ indicate the s-triazine ring structure of g-C_3_N_4_, which is evidence of proper phase formation [61]. In Figure 2, the FTIR spectra of the Co_3_O_4_ absorption spectra band at 3416 cm^−1^ and 1635 cm^−1^ show O-H stretching and bending vibrations, respectively. It might be due to moisture absorption of the sample from the environment [62, 63]. Furthermore, the absorbance bands from the FTIR spectra at 654 cm^−1^ and 558 cm^−1^ indicate vibrations of Co(III)-O bonds and Co-O stretching, confirming the formation of Co_3_O_4_ [60, 64, 65]. In Figure 2, the FTIR spectra of the ZnO absorption spectra band at 3400 cm^−1^ and 1380 cm^−1^ show O-H stretching and bending vibrations, respectively. It might be due to moisture absorption of the sample from the environment [66]. The absorbance bands from the FTIR spectra at 1110 cm^−1^ indicate vibrations of the Zn–O bond, confirming the formation of ZnO [67]. The FTIR results support the g-C_3_N_4_, ZnO and Co_3_O_4_ phase formation as claimed in XRD analysis. The FTIR spectra of the GZC-3 composite include bands associated with g-C_3_N_4_, Co_3_O_4_, and ZnO functional groups, respectively, confirming the successful synthesis of the g-C_3_N_4_-ZnO-Co_3_O_4_ composite as illustrated in Figure 2.

### 3.3. SEM Analysis

**Figure 3:**
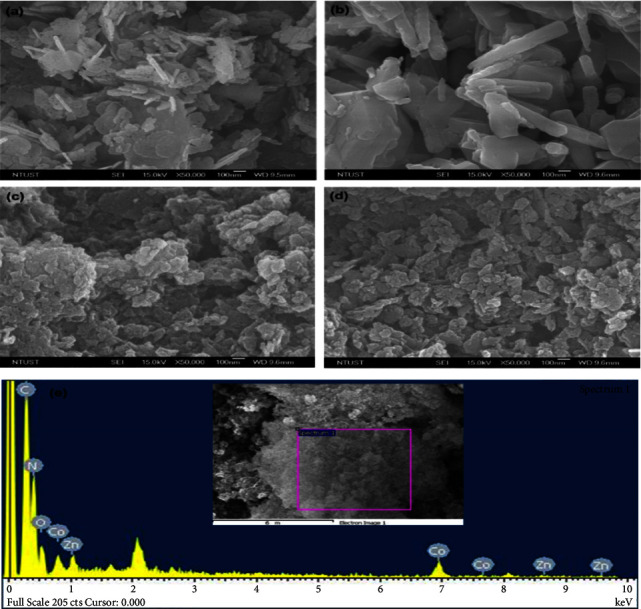
The SEM image of (a) Co_3_O_4_, (b) ZnO, (c) g-C_3_N_4_, (d) GZC-3 composite, and (e) EDX for the g-GZC-3 composite.

Scanning electron microscopy was used to examine the morphology of synthesized samples. Cellulose templates can bind metal cations and regulate the particle size of metal oxide during the synthesis process. When cobalt nitrate and zinc nitrate are mixed in a cellulose solution, the cobalt nitrate and zinc nitrate hydrolyze and interact to generate a strong adhesion on the cellulose surface because cellulose is rich in hydrogen bonds and a hydroxyl macromolecule. The physical space from cellulose makes it difficult for the formation of a large aggregation during the growth of these metal oxides. After calcination, the template is removed, resulting in dispersed ZnO and Co_3_O_4_. Figures 3(a) and 3(b) show the image of the SEM for the ZnO and Co_3_O_4_ grown using cellulose as a template, which has a characteristic of rod-like morphology. The rod-like morphology allows for faster kinetics of charge carriers to the catalyst's surface, which may reduce electron and hole recombination rates [68].  Figure 3(c) displays a sheet-like morphology for g-C_3_N_4_; it is typical for g-C_3_N_4_ synthesized by thermal polymerization followed by chemical exfoliation.  Figure 3(d) shows the image of the scanning electron microscope of the GZC-3 composite and illustrates the typical morphology of ZnO and Co_3_O_4_ across the matrix of g-C_3_N_4_. The chemical composition of the GZC-3 composite was analyzed using EDX spectroscopy, with the findings shown in Figure 3(e). The EDX spectra of the GZC-3 composite contained peaks corresponding to the Zn, Co, O, C, and N elements. These results clearly show the successful formation of the GZC composite.

### 3.4. UV Analysis

**Figure 4:**
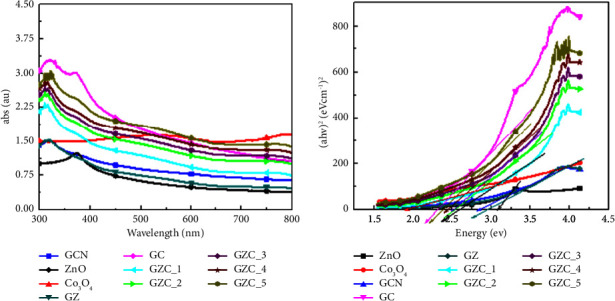
(a) Light absorption spectra of prepared catalytic samples. (b) The Tauc plot of g-C_3_N_4_, GZ, GC, GZC-1, GZC-2, GZC-3, GZC-4, and GZC-5 composites.

The optical absorbance of synthesized samples g-C_3_N_4_, ZnO, Co_3_O_4_, and GZC composite samples were measured by using a UV-vis spectrometer, as shown in Figure 4(a). ZnO absorbs more in the UV range of the solar spectrum, less than 400 nm wavelength, as shown in the spectra designated by the black line in Figure 4(a) [69]. The Co_3_O_4_ exhibited a strong absorption tail at a wavelength longer than 500 nm, as shown in the spectra designated by the red line in Figure 4(a) [70]. The *g*-C_3_N_4_ displayed high absorption capabilities between 250 and 380 nm, with an absorption edge at 460 nm, which is still short for absorbing the visible portion of solar radiation and leaving the large part of the visible light spectrum unexplored. The addition of Co_3_O_4_ and ZnO samples in the g-C_3_N_4_ matrix increased absorption intensity while also red-shifting the wavelength towards the visible light area (shifted to lower energy). The appropriate form of a junction formed between g-C_3_N_4_, ZnO, and Co_3_O_4_ might be the reason for the better absorbance to the visible area of the composites. The Tauc equation was employed to determine the energy bandgap of the synthesized samples from UV-visible absorbance spectra, as shown in Figure 4(b). The calculated optical band gaps of the prepared photocatalyst sample use Tauc's plots 3.1, 1.9, 2.85, 2.95, 2.3, 2.62, 2.55, 2.5, 2.4, and 2.35 eV for ZnO, Co_3_O_4_, g-C_3_N_4_, GZ, GC, GZC-1, GZC-2, GZC-3, GZC-4, and GZC-5 composites, respectively. The energy band gap of the composite samples seems to obey the superposition principle, as shown in Figure 4(b).

### 3.5. BET Surface Area and Pore Size Analysis

**Figure 5:**
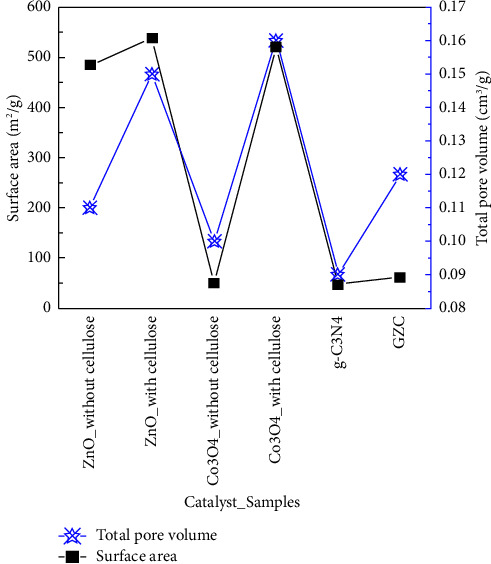
The specific surface area and total pore volume of samples.

The pore volume and the specific surface area of metal oxide prepared by cellulose templated or not are summarized in Figure 5. The specific surface area of ZnO prepared without cellulose measured 484.707 m^2^/g compared to cellulose templated 538.563 m^2^/g. The surface area of cobalt oxide prepared without cellulose is 48.673 m^2^/g, and with cellulose templated, it is 520.102 m^2^/g. For cellulose-templated ZnO and Co_3_O_4_, the total pore volume was 0.15 cm^3^/g and 0.16 cm^3^/g, respectively, whereas they were 0.11 cm^3^/g and 0.10 cm^3^/g for ZnO and Co_3_O_4_ without cellulose.

The results show that using cellulose as a template to prepare ZnO and Co_3_O_4_ result in higher total pore volume and specific surface area. The pore volume and specific surface area increment attributed to high-temperature cellulose decomposition and the creation of a porous structure of ZnO and Co_3_O_4_. The bulk g-C_3_N_4_ synthesized without the exfoliation process typically shows a poor surface area (<10 m^2^/g^−1^) [71]. The sulfuric acid-treated g-C_3_N_4_ surface area and pore volumes show 47.136 m^2^/g^−1^ and 0.09 cm^3^/g, respectively. The surface area of the GZC-3 composite (60.578 m^2^/g) and the pore volume (0.12 cm^3^/g) increased from bulk g-C_3_N_4_, as shown in Figure 5.

### 3.6. Photocatalytic Performance

The photocatalytic MB dye degradation process takes place in the photoreactor with a 250 Watts halogen lamp as the light source. Typically, around 30 mg of the prepared sample is added to a 100 ml (10 ppm) aqueous solution of MB dye. The catalyst-containing solution was stirred for 30 min in the dark before the photocatalytic degradation test commenced. That leads to achieving equilibrium for the adsorption and desorption of dye molecules on the photocatalyst surface, a dark reaction. To separate the solution from the photocatalyst powder, a 4 ml dye solution was taken and centrifuged to separate the catalyst and solution at each time interval. A UV-Vis spectrophotometer was used to measure the concentration of MB dye at each time interval. The distance between the light source and the solution remained constant at 5 cm. The MB dye degradation efficiency (*η*) was determined using the following formula: *η* = ((*C*_o_ − *C*_*t*_)/Co) ∗ 100%. The *C*_o_ and *C*_*t*_ are the MB dye concentrations at the initial and each time interval of light irradiation, respectively. All tests have been conducted again to validate the findings.

### 3.7. Photoluminescence (PL) Analysis

**Figure 6:**
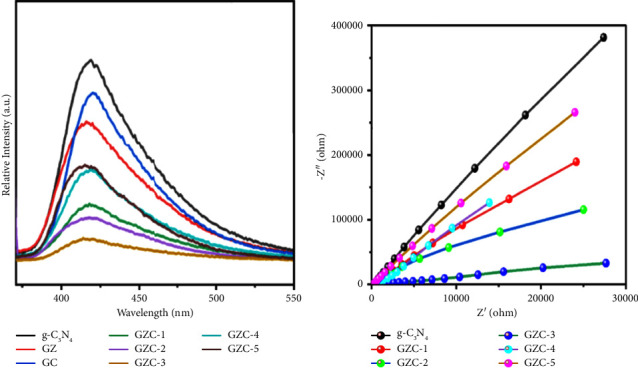
(a) Photoluminescence emission spectra of g-C3N4, GZ, GC, GZC-1, GZC-2, GZC-3, GZC-4, and GZC-5 and (b) electrochemical impedance spectroscopy of g-C3N4, GZC-1, GZC-2, GZC-3, GZC-4, and GZC-5.

To observe the recombination of photogenerated charge carriers in the catalyst room temperature, PL spectra were measured at 326 nm excitation wavelength, as shown in Figure 6(a). The g-C_3_N_4_ sample has the maximum PL peak intensity and exhibits wider peak width emission at 419 [43], demonstrating a high rate of electron and hole recombination relative to the synthesized sample. The formation of GZC composites results in photoluminescence quenching; it might boost photocatalytic activity.  Figure 6 illustrates the PL emission spectra of the photocatalysts g-C_3_N_4_, showing the highest peak and the composite GZC's lowest peak. The peak intensity is from highest to lowest in the order *g*-C_3_N_4_, GC, GZ, GZC-5, GZC-4, GZC-1, GZC-2, and GZC-3, as shown in Figure 6(a). The GZC-3 composite shows the lowest PL emission peak intensity among all synthesized catalysts, implying the effective suppression of photogenerated charge carriers from recombination. This phenomenon will favor having a large number of electrons and holes engage in the redox process.

### 3.8. The Nyquist Plots

Electrochemical impedance spectroscopy (EIS) is a powerful technique for understanding the transport of interface charges [72]. We examined the charge transfer resistances and interfacial charge separation efficiency of *g*-C_3_N_4_ and GZC composites by EIS, as shown in Figure 6(b). During interfacial transport, the composites possess the smallest EIS semicircle radius compared to g-C_3_N_4_, implying the lowest impedance, which permits the rapid transfer of charges. Based on the EIS data, the GZC-3 composite catalyst has shown a small radius compared to other samples in this experiment. Small radii correspond to small charge transfer resistance, thereby increasing the efficiency of photocatalysts, which complement the PL spectra somewhere above.

### 3.9. Photocatalytic Activity of the Samples

**Figure 7:**
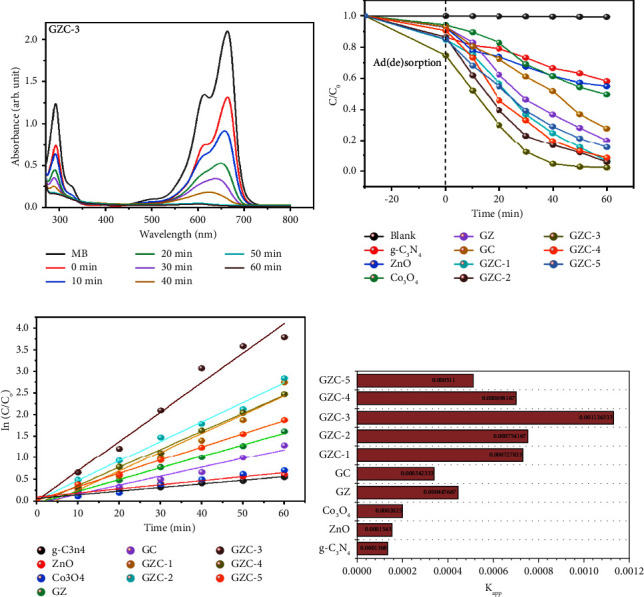
(a) UV absorbance of MB dye at different time intervals for the GZC-3. (b) First-order kinetics in MB dye. (c) Logarithm versus time of g-C_3_N_4_, ZnO,Co_3_O_4_, GZ, GC, GZC-1, GZC-2, GZC-3, GZC-4, and GZC-5. (d) Degradation rate constant *k* (min^−1^) of the as-prepared samples against g-C_3_N_4_, GZ, GC, GZC-1, GZC-2, GZC-3, GZC-4, and GZC-5.

The photocatalytic MB dye degradation performance of the synthesized samples was measured using the beer lambert law. The catalyst-dye-containing solution was irradiated, and the dye concentration was measured by UV-visible spectroscopy. The absorbance vs. wavelength graph of the GZC-3 composite catalyst change is shown in Figure 7(a), and the concentration of MB dye over time changed in the presence of the catalyst.

The blank test indicates that MB dye is only weakly degraded in the absence of a catalyst, noting that photolysis is not an option to degrade the MB dye, as shown in Figure 7(b) gray line. The g-C_3_N_4_, ZnO, and Co_3_O_4_ exhibited photocatalytic degradation efficiency for the degradation of MB dye 41.92, 45.10, and 50.40% of MB, respectively. The binary and ternary composites of g-C_3_N_4_, ZnO, and Co_3_O_4_ exhibit improved photodegradation activity for MB dye degradation. In particular, the ternary composites g-C_3_N_4_/ZnO/Co_3_O_4_ show promising performance for the photodegradation of MB. The GZC-3 composite had the maximum photodegradation activity showing 97.4% MB dye degradation after 60 min of photoreaction.

The degradation kinetic was studied to highlight the photodegradation activity of the materials, as shown in Figure 7(c). The pseudo-first-order kinetic equation -ln (*C*_*t*_/C_0_) = *k*_app_ was used to fit the degradation kinetic plots, where *k*_app_ and *t* represent the apparent first-order reaction rate constant and irradiation time, respectively.  Table 1 shows the corresponding correlation coefficient values, which are all close to one, showing that the photocatalytic degradation process is a first-order reaction. On the other hand, observing the results of the kinetic plot in Figure 7(d), the GZC-3 composite has the highest value of *k*_*app*_ (0.001136 min^−1^); in comparison, the *k*_app_ values for g-C_3_N_4_, ZnO, and Co_3_O_4_ are, respectively, 0.000137, 0.000154, and 0.0002025 min^−1^. It implies that the photodegradation activity of the GZC-3 composite is approximately 8.29, 7.38, and 5.60 times greater than pure g-C_3_N_4_, ZnO, and Co_3_O_4_, respectively.

### 3.10. Recyclability

A catalyst for practical use has to examine its photostability in the effluent. The GZC-3 composite catalyst sample measured the photocatalytic MB dye degradation activity for 60 min. Subsequently, 4.0 ml of the degraded solution has taken to measure the dye concentration, and the remaining degraded solution was centrifuged and separated the catalyst from the solution. The separated catalyst was washed and cleaned with distilled water and dried in an electric oven at 60°C for 2 hrs. The recyclability and photostability of the catalyst were measured for four cycles following the procedure.

**Figure 8:**
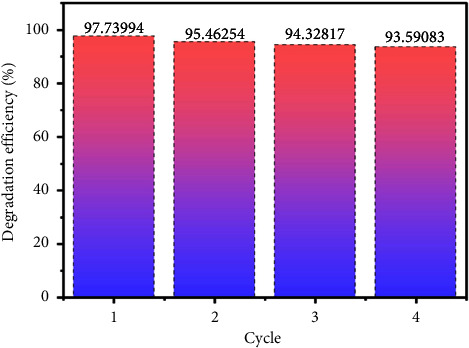
The recyclability test of the GZC-3 composite.

The catalyst shows excellent stability in degrading MB dye in an aqueous solution, as shown in Figure 8 and Figure S7. However, after the four cycles of the degradation process, negligible fluctuation in the degradation performance was observed, which might be attributed to the loss of catalyst amount while collecting from the centrifuge tube, since the amount of the catalyst added in the dye solution is small.

### 3.11. Reaction Mechanism

**Figure 9:**
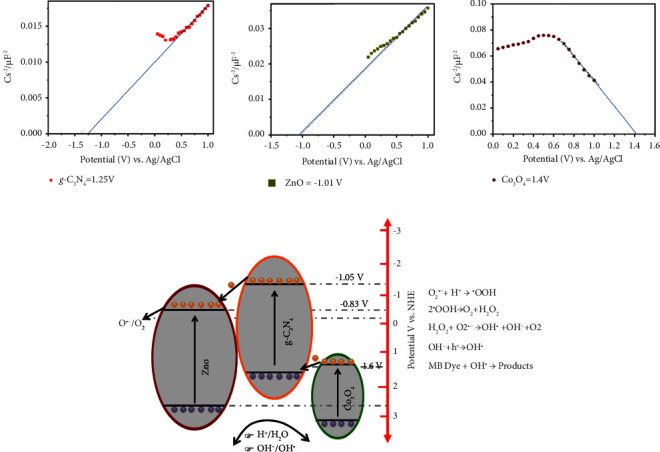
(a–c) Mott–Schottky plots of g-C_3_N_4_, ZnO, and Co_3_O_4_, respectively. (d) The possible photocatalytic degradation mechanism of the g-C_3_N_4_/ZnO/Co_3_O_4_ composite against MB.

The flat band potential of the composite materials was measured using the Mott–Schottky technique at 800 Hz in the dark, as shown in Figures 9(a)–9(c). The Mott–Schottky plot for the g-C_3_N_4_ and ZnO shows a positive slope, which indicates *n*-type conductivities. The Co_3_O_4_ has a negative slope indicating *p*-type conductivities.

Extrapolating the linear section of the plot to the *x*-axis shows the flat band potentials (V_FB_) of g-C_3_N_4_, ZnO, and Co_3_O_4_ as −1.05 V, −0.83 V, and 1.6 V vs. RHE, respectively. The conduction band edge potential of g-C_3_N_4_, ZnO, and Co_3_O_4_ was approximately the same as the V_FB_ [73]. The valence band edge position of g-C_3_N_4_, ZnO, and Co_3_O_4_ is determined to be ∼1.7 V, 2.3 V, and 3.4 V vs. RHE; therefore, the composite may create a junction band structure shown in Figure 9(d).

Up on irradiation to the catalyst in the dye solution, electrons from the valance bands of g-C_3_N_4_, ZnO, and Co_3_O_4_ are excited to the conduction band of g-C_3_N_4_, ZnO, and Co_3_O_4_, which creates a large number of charge carriers in the catalyst valance and conduction band. The electron on the conduction band of cobalt oxides will fall to the valance band of g-C_3_N_4_ due to the potential variance; this could result in a Z-scheme electron transfer process [74, 75], as shown in Figure 9(d).

The excited electron on the conduction band of g-C_3_N_4_ will transfer to the conduction band of the ZnO. The Z-scheme improves the photogenerated charge carriers' transfer and separation efficiency while maintaining the strong reduction capability of electrons in g-C_3_N_4_ transferred to the ZnO and oxidation capability of holes in the ZnO and Co_3_O_4_ for effective MB dye degradation.

The electron in the conduction band of ZnO will participate in the reduction process to produce superoxide (O^•−^) radicals, and the holes in the valance bands of ZnO and Co_3_O_4_ will primarily participate in the oxidation reaction to form hydroxyl (OH^•^) radicals.

ZnO is thermodynamically more feasible to undergo a reduction reaction to produce superoxide (O^•−^) radicals due to high negative conduction band potential (−0.8 vs. NHE) compared to (O^•−^/O_2_), whereas the hole in the ZnO and Co_3_O_4_ valance band are thermodynamically more feasible to undergo oxidation process to hydroxyl (OH^•^) radicals due to sufficient positive valance band potentials compared to H^+^/H_2_O and OH^−^/OH^•^ [76–78]. This active species (O^•−^) and OH• reacts with MB dye to break down the bond of the dye into nonpolluting molecules such as water, carbon dioxide, and others.

## 4. Conclusions

The Z-scheme ZnO-g-C_3_N_4_-Co_3_O_4_ composite was prepared by wet chemical and ultrasonicate-assisted synthesis methods. It has been observed that utilizing cellulose as a template to prepare metal oxide increases the specific surface area with a distinct morphology that might enhance the density of the active site. The photocatalytic activity of the GZC composite towards MB dye degradation was better than that of the pure and binary composites. The GZC-3 composite demonstrated the highest degradation efficiency in degrading MB dye, with 97.4% degradation efficiency in 60 min under visible light irradiation. All the synthesized composite photocatalysts exhibit improved photocatalytic activity compared to the binary composite and pure ones due to the proper band alignment, the higher specific surface area, better visible light absorbance, and low charge transfer resistance. The GZC-3 composite demonstrates excellent stability and degradation efficiency, suggesting that GZC is a feasible candidate photocatalytic material for MB dye degradation in an aqueous solution.

## Figures and Tables

**Table 1:** Efficiency, *K*_app_, and *R*^2^ values of the catalytic samples.

Samples	Efficiency (%)	*K*_app_ (min^−1^)	Correlation coefficient (*R*^2^)
g-C_3_N_4_	41.92	0.000137	0.94606
ZnO	45.1	0.000154	0.91847
Co_3_O_4_	50.4	0.0002025	0.99151
GZC-1	93.56	0.000728	0.95585
GZC-2	94.16	0.000754	0.99036
GZC-3	97.40	0.001136	0.97613
GZC-4	91.54	0.000698	0.99648
GZC-5	84.64	0.000511	0.99774
GC	72.35551	0.000342	0.96373
GZ	80.01815	0.000448	0.99567

## Data Availability

The data used to support the findings of this study are included within the article, and the supporting information/supplementary file is available from the Hindawi Online Library.

## Supplementary Materials

Supplementary Materials Figure S1: schematic illustration of the celluloses synthesis process. Figure S2: schematic illustration of the ZnO synthesis process. Figure S3: schematic illustrations of the Co_3_O_4_ synthesis process. Figure S4: schematic illustration of the g-C_3_N_4_ synthesis process. Figure S5: schematic illustration of the g-C_3_N_4_/ZnO/Co_3_O_4_ composite synthesis process. Figure S6: XRD of prepared cellulose.
