# Supplementary material for: g-C3N4–Co3O4 Z-Scheme Junction with Green-Synthesized ZnO Photocatalyst for Efficient Degradation of Methylene Blue in Aqueous Solution
Source: Bioinorg Chem Appl. 2023 Jun 5;2023:2948342. doi: 10.1155/2023/2948342 (PMC10260312; doi:10.1155/2023/2948342)
Supplement: Supplementary Materials — Figure S1: schematic illustration of the celluloses synthesis process. Figure S2: schematic illustration of the ZnO synthesis process. Figure S3: schematic illustrations of the Co3O4 synthesis process. Figure S4: schematic illustration of the g-C3N4 synthesis process. Figure S5: schematic illustration of the g-C3N4/ZnO/Co3O4 composite synthesis process. Figure S6: XRD of prepared cellulose. [file 2948342.f1.docx]

Supporting Information

Z-scheme Co3O4−g-C3N4−ZnO Heterojunction Photocatalyst for Efficient Degradation of Methylene Blue in Aqueous Solution

**Mintesinot Tamiru Mengistu^1^, Tadele Hunde Wondimu^1,2^, Dinsefa Mensur Andoshe^1*^, Jung Young Kim^1,2^ Osman Ahmed Zelekew^1^, Fekadu Gashaw Hone^3^, Newaymedhin Aberra Tegene^3^, Noto Susanto Gultom^4^, Ho Won Jang^5^**

^1^Department of Materials Science and Engineering, Adama Science and Technology University, Adama, PO BOX 1888, Ethiopia.

^2^Center of Excellence (COE) for Advanced Manufacturing Engineering, Adama Science and Technology University, Adama, Ethiopia.

^3^Physics Department, Addis Ababa University, Addis Ababa, 1176, Ethiopia,

^4^Department of Materials Science and Engineering, National Taiwan University of Science and Technology, Taipei 10607, Taiwan,

^5^Department of Materials Science and Engineering Research Institute of Advanced Materials Seoul National University, Seoul 08826, Republic of Korea,

*Correspondence: [dinsefa.mensur@astu.edu.et](mailto:dinsefa.mensur@astu.edu.et)


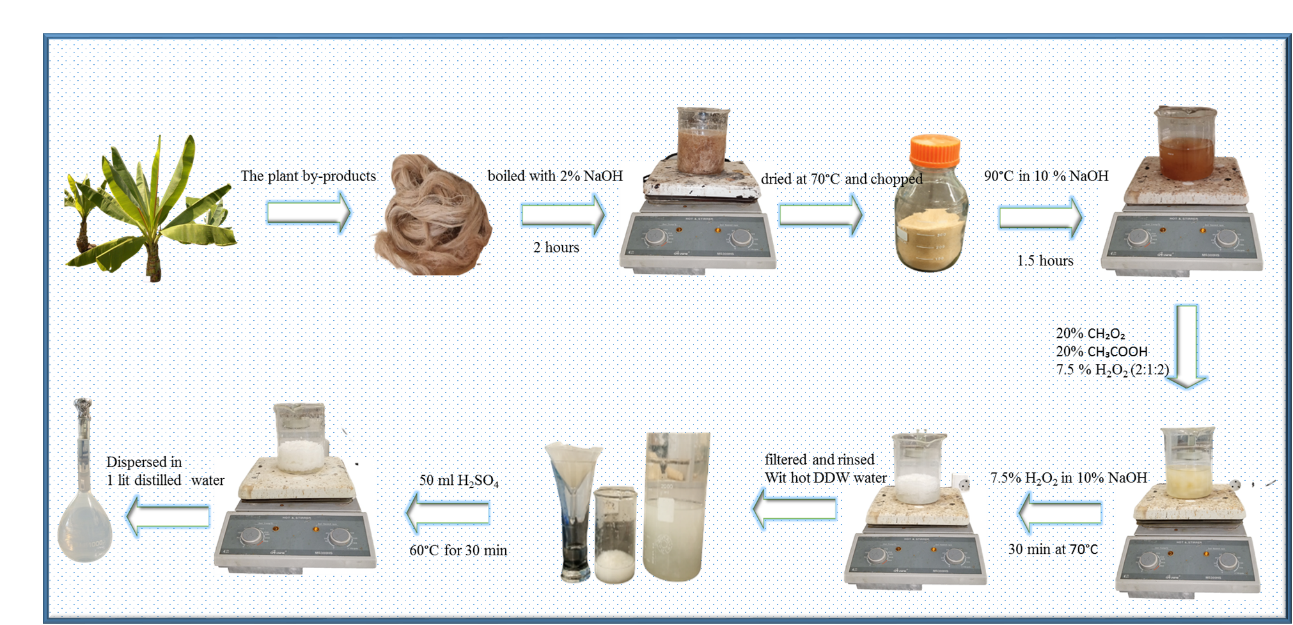


Fig. S1 Schematic illustration of the celluloses Synthesis process.


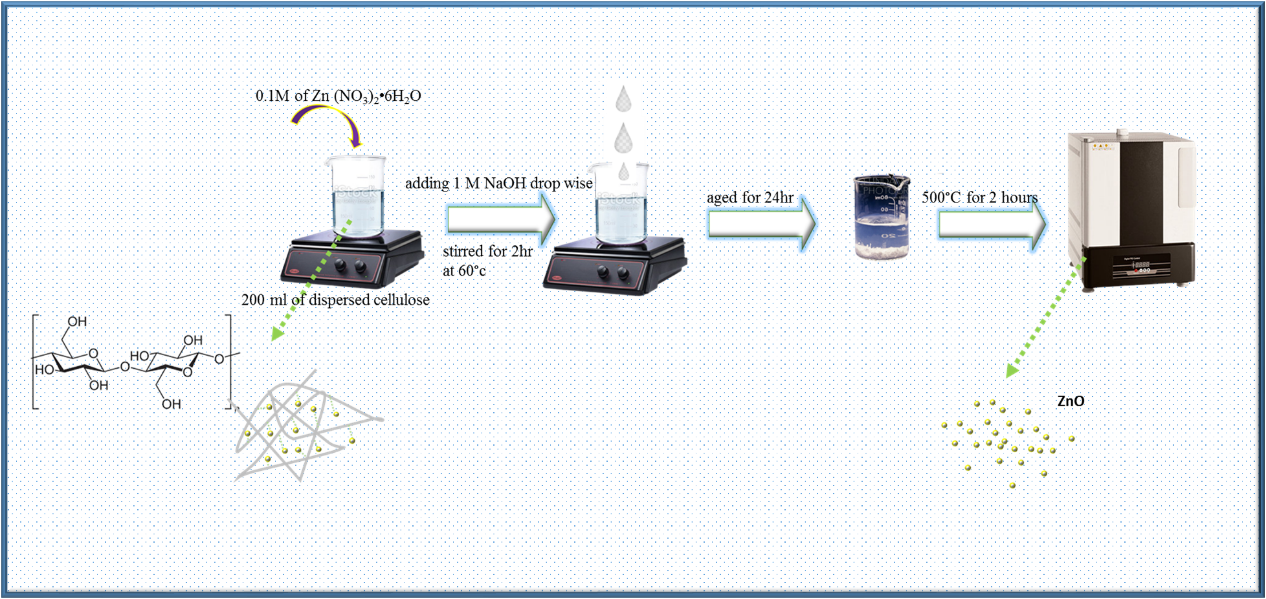


Fig. S2 Schematic illustration of ZnO Synthesis process.


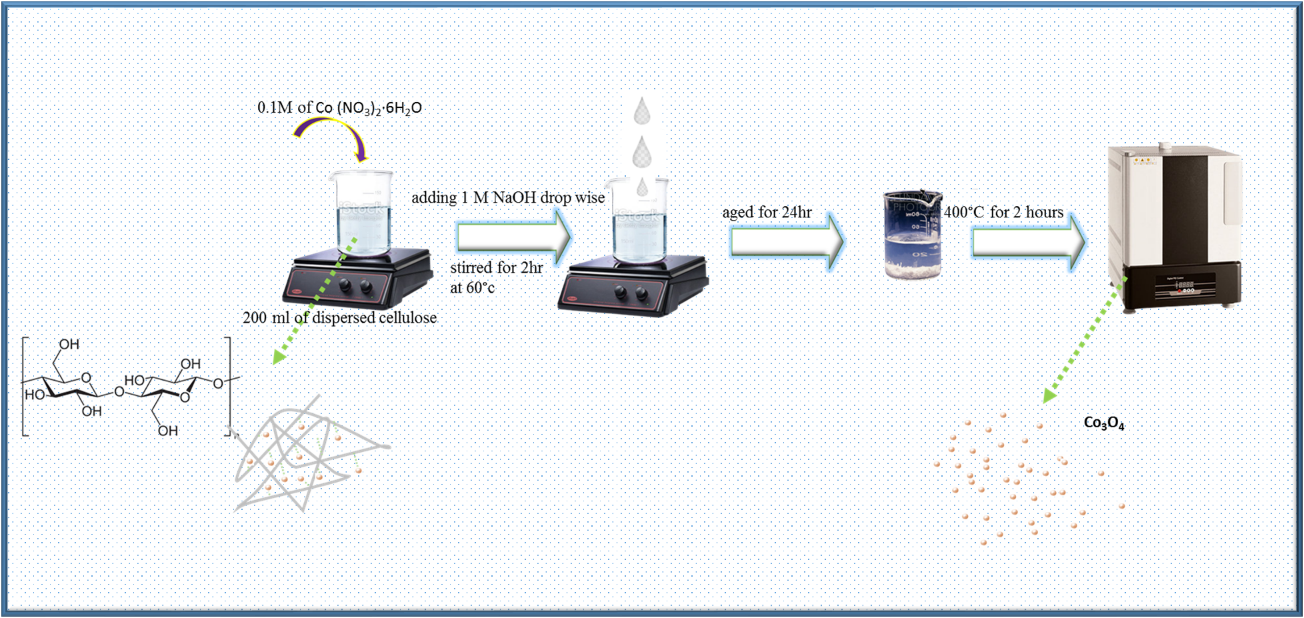


Fig. S3 Schematic illustrations of the Co_3_O_4_ Synthesis process.


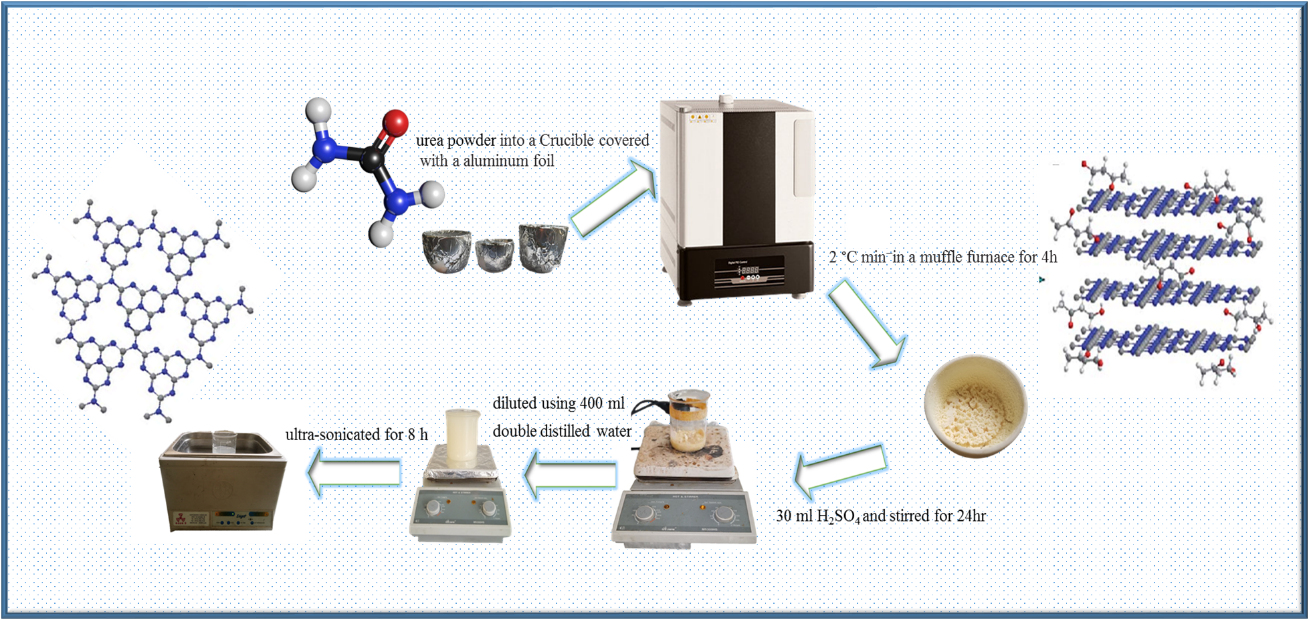


Fig. S4 Schematic illustration of the g-C_3_N_4_ Synthesis process.


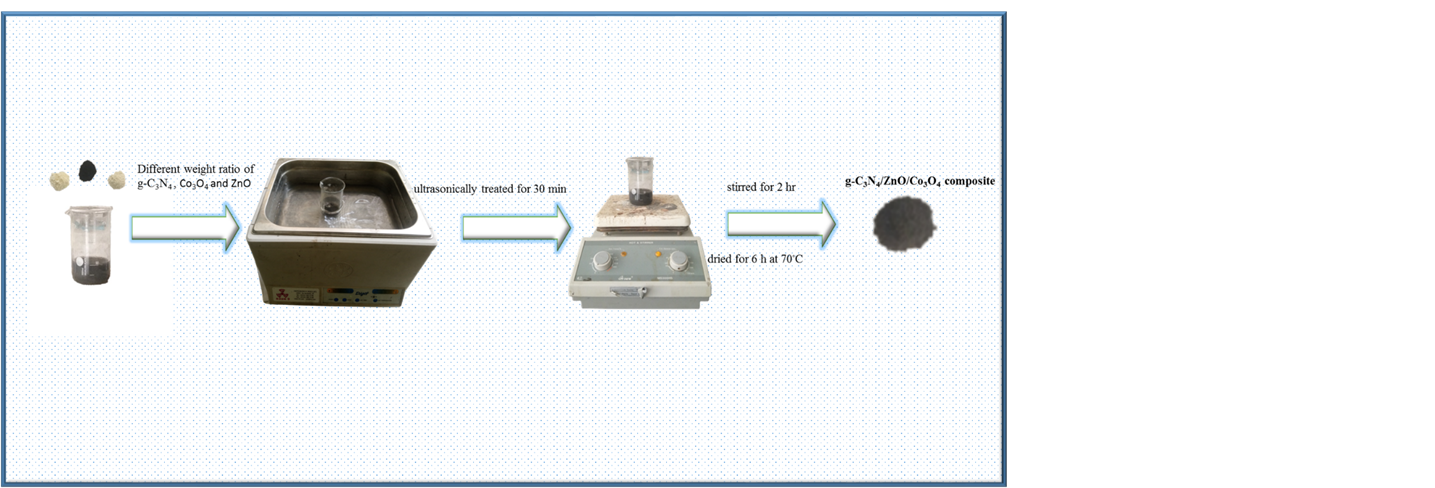


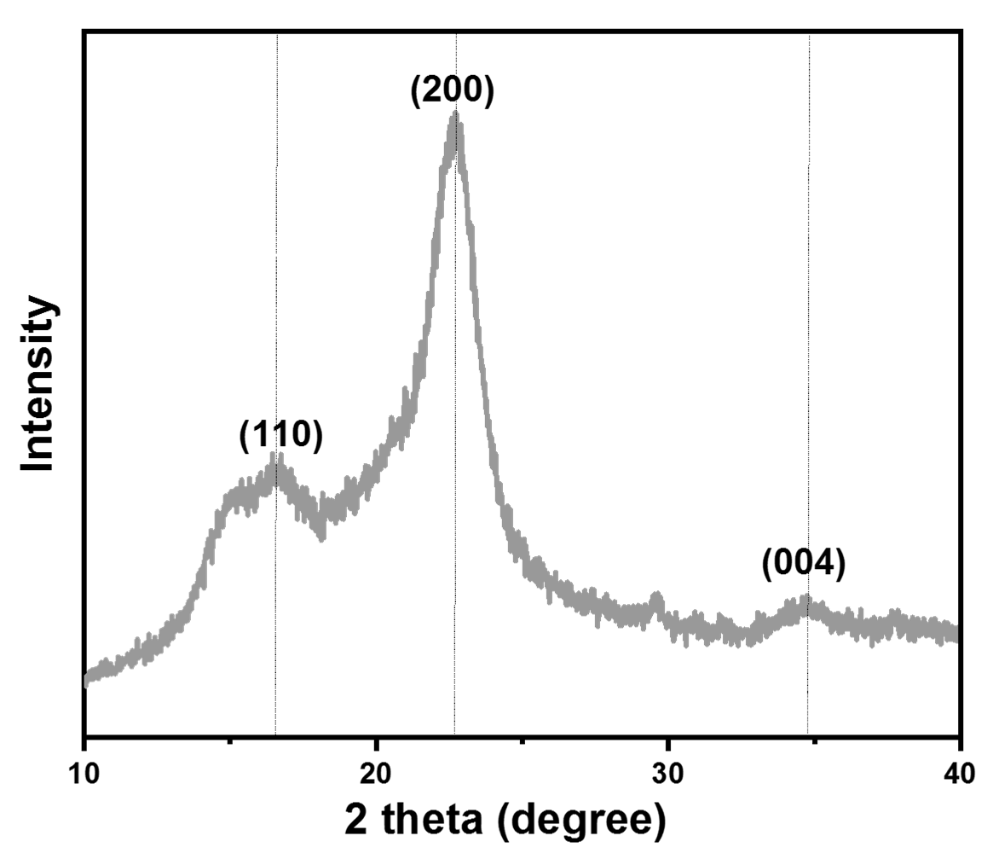
Fig. S5 schematic illustration of g-C_3_N_4_/ZnO/Co_3_O_4_ composite Synthesis process.

Fig. S6 XRD patterns of cellulose.

**
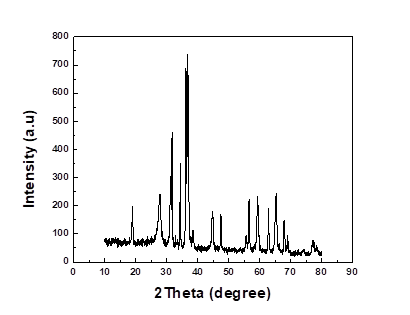
**

**Fig S 7. XRD for the GZC-3 after stability test measurement**
